# Inflammatory breast cancer, best practice in the community setting

**DOI:** 10.1038/s41523-025-00765-4

**Published:** 2025-06-07

**Authors:** Yoko Takahashi, Nithya Sridhar, Toshiaki Iwase, Ashley Marumoto, Jami Fukui, Aiesha Pradhan, Yee Chung Cheng, Naoto T. Ueno

**Affiliations:** 1 University of Hawaiʻi Cancer Center Inflammatory Breast Cancer Clinic and Research Program, Honolulu, HI USA; 2 Translational and Clinical Research Program, University of Hawaiʻi Cancer Center, Honolulu, HI USA; 3 Department of Internal Medicine, Baylor College of Medicine, Houston, TX USA; 4 Department of Surgery, University of Hawai’i John A. Burns School of Medicine, Honolulu, HI USA; 5 Department of Science and Technology Studies, Cornell University, Ithaca, NY USA; 6 Division of Hematology and Oncology, Department of Medicine, Froedtert and Medical College of Wisconsin, Milwaukee, WI USA

**Keywords:** Breast cancer, Breast cancer

## Abstract

Inflammatory breast cancer (IBC) is a rare and aggressive form of breast cancer typically diagnosed at advanced stages. Although many cases initially respond to conventional therapies, IBC remains refractory, with high risk of recurrence due to early dissemination, tumor heterogeneity, and complex microenvironmental factors. Despite advancements in treatment, IBC poses unique challenges, particularly in community healthcare settings, where implementation of current guidelines is often limited by disease complexity and evidence gaps. Multidisciplinary care is essential and should include education on therapeutic options, lymphedema management, financial navigations, and ongoing support. To support diagnostic consistency, a consensus-driven IBC Scoring System has been developed to help clinicians identify IBC more accurately using clinical features This paper reviews best practices for managing IBC in community settings, emphasizing practical, multidisciplinary strategies that improve outcomes and presenting a framework aligns with the realities of community healthcare to ensure patients receive the highest possible standard of care.

## Introduction

Inflammatory breast cancer (IBC) represents 2-6% of all breast cancer diagnoses in the United States and is known for its aggressive behavior and poor prognosis^1,2^. Despite numerous efforts over the decades to improve the clinical outcomes, the prognosis for IBC remains worse compared to non-IBC. Approximately 30-40% of newly diagnosed IBC patients have distant metastasis, and the historically reported five-year survival rate is around 40%^3^. The diagnosis of IBC relies on a constellation of clinical manifestations, including rapid onset of erythema, edema, and *peau d’orange*, corroborated by the American Joint Committee on Cancer’s 8th edition definition of T4d malignancies. Histological confirmation through skin biopsy is helpful but not required. Recently, a novel scoring system^4^ (Table 1) has been adopted to mitigate the reliance on subjective clinical experience, which has historically guided diagnoses.

**Table 1:** Suggested scoring system for diagnosing inflammatory breast cancer

Characteristic	Score3	Score2	Score1	Priority factor (multiplier)
Timing of signs/symptoms	≤3 months	3–6 months	>6 months	×3
Skin changes	Any peau d’orange	Skin edema/thickening* ver ≥ 1/3 of the breast	Focal skin edema/thickening* (<1/3 of the breast)	×3
Swelling/engorgement of the breast	Clinically apparent enlargement of the breast or new asymmetry in breast size	*Intentionally blank; patients receive either a score of 3 or 1 for this characteristic*	Breast edema identified on imaging but not clinically detectable	×3
Erythema or other skin discoloration: pink, red, darkened, bruising/purplish, or serpiginous in character	Complete or near complete involvement of the breast	Not nearly complete but greater than minimal involvement of the breast	Minimal involvement or ambiguous color change	×2
Nipple abnormalities	New nipple inversion	New nipple flattening or other asymmetry	Crusting of the nipple/areola without other nipple changes	×2
Lymphatic tumor cell emboli	Dermal lymphatic emboli present (without evidence of direct involvement of the dermis or epidermis)	Non-dermal lymphatic emboli present (breast parenchyma or stroma)	*Intentionally blank; patients receive either a score of 3 or 2 for this characteristic*	×2
Breast imaging	Diffuse involvement of breast parenchyma (with or without dominant mass)	*Intentionally blank; patients receive either a score of 3 or 1 for this characteristic*	Enlargement of non-axillary nodes (internal mammary, supraclavicular, subpectoral, etc.)	×1

Adapted from Jagsi R, et al. Breast Cancer Res Treat. 2022;192(2):235–243. Licensed under CC BY 4.0. 10.1007/s10549-021-06434-x.

Clinical, pathologic, and imaging characteristics are listed in rows with a graded scoring system of 1–3 listed in columns, where 3 is definitively associated with inflammatory breast cancer and 1 less specific for IBC compared with non-inflammatory locally advanced breast cancer. If a characteristic it is totally absent, enter a score of zero. If statements in multiple columns describe the patient presentation, the highest scoring column should be selected. The priority factor in the far-right column represents how some characteristics are more heavily weighted for inflammatory breast cancer and thus represent a multiplying factor. The score for each characteristic is multiplied by the priority factor, then the subtotals of all the characteristics are added together to yield a total score (Total IBC Value). The Total IBC Value provides a score for use in identifying inflammatory breast cancer. Boxes marked “Intentionally blank” are not factored into the score. Proposed classifications of the Total IBC Scores are DEFINITELY IBC (total score ≥ 42); STRONG POSSIBILITY of IBC (total score 25–41); WEAK POSSIBILITY of IBC (total score 14–24); and NOT IBC (total score < 14).

*Skin thickening may be assessed on clinical examination or observed on breast imaging.

In terms of treatment, the absence of randomized controlled trials specific to IBC has resulted in an indirect extrapolation of data from trials predominantly involving non-IBC participants. Therefore, the treatment for newly diagnosed IBC is similar to other types of locally advanced breast cancers. It consists of neoadjuvant systemic therapy, surgery, and radiation therapy. IBC has no specific molecular marker, making developing IBC-specific therapies challenging. There are IBC-specialized clinics (MD Anderson Cancer Center, Dana Farber Cancer Institute, University of Hawaiʻi Cancer Center, etc.). However, given the limited availability of specialized IBC clinics, this review is meticulously crafted to distill actionable insights from the collective expertise of IBC specialists.

The increasing emphasis on “holistic patient” care necessitates comprehensive management of IBC, including mitigating the adverse effects of therapies and improving patients’ quality of life^5^. Integrating a multidisciplinary cancer care team has become indispensable in delivering optimal care and treatment to IBC patients. This review aims to coalesce recent findings regarding the diagnosis and management of IBC and propose a multidisciplinary IBC management framework to ensure effective patient care.

## Methods

The search terms “Inflammatory breast cancer,” “breast cancer,” “neoadjuvant,” “clinical trial,” “Breast cancer-related lymphedema,” and “ctDNA” were used to retrieve relevant publications from PubMed, as well as presentations from major conferences and congresses, including the American Society of Clinical Oncology (ASCO) Annual Meeting, the Breast Cancer Symposium, and the San Antonio Breast Cancer Symposium. We also searched the Guidelines from the National Comprehensive Cancer Network (NCCN) and clinical trials listed on ClinicalTrials.gov. We then assessed all collective information for their relevance to inflammatory breast cancer. Finally, we described the results based on answering the common essential questions about IBC management posed by the community clinical setting.

### Essential questions related to IBC

#### How do we recognize IBC?

The first step of the clinical practice of IBC is to make an accurate diagnosis. Due to its rarity and ambiguous presentation, inaccurate diagnoses and misdiagnoses are common^6^. Prompt and accurate diagnosis and treatment are crucial due to the aggressive nature of the disease^3^. IBC symptoms can vary significantly between individuals. In addition to the rapid onset (≤6 months) of breast erythema, symptoms may include peau d’orange edema, swelling or enlargement of the breast, warmth to the touch, a flattened or retracted nipple, and swelling of lymph nodes in the axilla or around the collarbone^7,8^. It is important to note that experiencing these symptoms does not necessarily indicate IBC, nor does their absence rule it out. These symptoms may occur with or without an underlying palpable mass. IBC is typically diagnosed through a clinical exam and, like non-IBC breast cancer, confirmed with a breast biopsy. This distinct clinical syndrome, characterized by diffuse erythema and edema (*peau d’orange*) covering a third or more of the breast skin, poses diagnostic challenges that often overlap with cellulitis or mastitis. The 8th edition of the American Joint Committee on Cancer (AJCC) staging manual continues to define IBC based on the presence of diffuse erythema and edema involving a third or more of the breast after confirmation of invasive breast cancer. IBC uses the same staging system as non-IBC, and the disease is considered at least a stage IIIB cancer. The diagnosis of IBC is categorized as clinical stage T4d^9^.

Unlike most other breast cancers, IBC symptoms progress very quickly, often within days or weeks, and most patients with IBC never detect a lump. Increased awareness of IBC has led some patients to seek healthcare attention earlier, when clinical characteristics may not be less pronounced^10^. However, delays in diagnosis and referral for appropriate treatment are common due to the absence of pathognomonic hallmarks. Even experienced clinicians may struggle with diagnosis due to the variability in individual presentation. A study revealed that 38% of cases resulted in diagnostic errors, highlighting the urgent need for improved diagnostic methods^3,10^. Moreover, IBC’s clinical presentation can resemble mastitis and can be initially misdiagnosed. Mastitis often involves a bacterial infection treated with antibiotics, whereas IBC is not an infection and does not respond to antibiotics. If antibiotic treatment proves ineffective within a week of diagnosing mastitis, we should consider evaluating for IBC.

To address these challenges, Susan G. Komen for the Cure®, in collaboration with the IBC Research Foundation and the Milburn Foundation, convened a distinguished panel of experts dedicated to advancing research and care for IBC. This multidisciplinary team, consisting of oncologists, pathologists, radiologists, researchers, and patient advocates, aimed to refine the diagnostic and therapeutic approach to this aggressive and often misdiagnosed form of breast cancer. Their latest initiative resulted in the development of an advanced IBC Diagnostic Scoring System^4^ (Table 1), which has shown significant promise in reducing the diagnostic ambiguity that frequently complicates IBC cases (see next section).

Finally, the recent increase in awareness of IBC has led to cases where patients present with only slight redness, or sometimes no redness at all, but often with or without diffuse breast engorgement. This variability creates significant challenges in diagnosis, as these cases do not neatly fit into established criteria. Nonetheless, some patients exhibit diffuse breast edema detectable by MRI or mammography. In these cases, even a low-to-moderate Komen score may warrant a diagnosis of IBC. The lack of a clear, universally accepted definition of IBC impacts patient care and research, leading to subjective diagnoses and inconsistent treatments.

#### How can we accurately diagnose IBC?

Prompt and accurate diagnosis of IBC continues to be a significant challenge. To address this, Susan G. Komen for the Cure®, in collaboration with the IBC Research Foundation and the Milburn Foundation, assembled a panel of experts to develop a diagnostic scoring system by expert consensus (Table 1). This system evaluates clinical characteristics and variations in established conditions to provide a more consistent and accurate diagnosis of IBC^4^. The primary goal of the IBC Scoring System is to facilitate prompt, precise, and consistent identification of IBC in clinical settings.

The scoring system assesses several clinical features, each rated on a scale of 1 to 3 based on the severity or presence of symptoms. These features include the timing of signs/symptoms, skin edema or thickening, swelling/engorgement of the breast, erythema or other skin discoloration (such as pink, red, darkened, bruising/purplish, or serpiginous), nipple abnormalities, lymphatic emboli, and breast imaging. Given the dynamic nature of IBC, clinical breast exams may change over time, potentially affecting the total score. To ensure diagnostic accuracy, the score should reflect the highest level observed, even if specific symptoms are absent. A higher score correlates with a more classic and definitive diagnosis of IBC.

Recent validation using a large multi-institutional IBC clinical database demonstrated that the scoring system effectively discriminates between IBC (T4d) and non-IBC (T4b/c) patients. Given its ability to improve diagnostic consistency, the SGK IBC Scoring System should be recommended as a routine diagnostic approach in clinical practice. While its complexity may present a barrier to widespread use, it remains a valuable tool in cases of diagnostic uncertainty. Despite its limited specificity, it provides a structured framework that can guide evaluation and support treatment planning. Although not definitive on its own, its consistent use can reduce diagnostic delays and promote guideline-concordant care. Ongoing studies will further clarify its accuracy, reproducibility, and complementary role alongside existing diagnostic methods^11^.

In cases where clinical findings suggest IBC, a skin punch biopsy should be performed on the area showing the most significant changes, such as erythema or edema (peau d’orange). In particular, these biopsies are crucial if the redness extends to skin areas, which may impact operability determination; as with the AJCC diagnostic criteria, dermal lymphatic emboli are not required for diagnosis using this scoring system, provided other criteria are met, and a score of greater than 42 is documented. For patients with a ‘strong possibility of IBC,’ trimodality therapy—including preoperative systemic therapy, mastectomy, and radiation therapy—is recommended, following the same treatment approach as for confirmed IBC cases^12^. For those with a lower likelihood of IBC, treatment decisions should be based on disease phenotype, anatomical extent, and the patient’s individual goals. While trimodality therapy may be appropriate for some patients in this category, it should not be universally mandated.

Reliance on pathological findings alone is a limitation, as the complexity of IBC often necessitates a comprehensive, multidisciplinary approach to diagnosis. The IBC scoring system offers a promising advancement by reducing diagnostic ambiguity through a more structured and quantifiable method. However, many clinical factors remain subjective. If accurate, patients diagnosed with IBC using this scoring system should exhibit a different clinical course and outcome compared to those with locally advanced breast cancer (LABC) who do not meet the IBC definition. Ultimately, discovering and validating distinct molecular or genetic markers that can differentiate IBC from non-inflammatory LABC will be essential to eliminate the subjectivity in diagnosis. Once identified, these markers should be studied for their sensitivity and specificity, especially in patients with lower scores, to refine their diagnostic utility.

To further enhance the application of this scoring system, a new online tool has been made available to assist healthcare providers in recognizing and diagnosing IBC^13^. By providing the proposed IBC diagnostic criteria in a user-friendly format, the tool allows clinicians to identify IBC more quickly and effectively, potentially leading to earlier diagnoses and improved patient outcomes. The latest IBC scoring system shows promise in reducing diagnostic ambiguity by offering a structured, quantifiable method for diagnosis. While the reliance on pathological findings alone remains a limitation, the system represents a critical step forward in addressing the complexity of IBC, reinforcing the need for a comprehensive, multidisciplinary approach to diagnosis and treatment.

#### What imaging should we order to diagnose and manage IBC?

Imaging plays a critical role in diagnosing and managing IBC, particularly given its atypical presentation, which often lacks a distinct mass^14–16^. The majority of women with IBC present with locoregional disease, and up to 40% have distant metastases (Stage IV) at diagnosis^17,18^. Accurately determining the stage and extent of the disease is essential for effective treatment planning, including surgery and radiation therapy.

Medical photography is routinely performed before neoadjuvant chemotherapy (NAC) to visually document the extent of skin involvement, information crucial for planning future radiotherapy fields and surgical interventions.

Comparative analysis of mammograms and ultrasound images can reveal key indicators of IBC, such as skin thickening, trabecular thickening, increased breast density, and axillary adenopathy. For example, mammograms frequently reveal skin thickening (84-93%), increased breast density (93%), and axillary adenopathy (24%)^17,19^. Ultrasound is valuable for detecting skin thickening, hypoechoic non-mass abnormalities, calcifications, and nodal disease. Moreover, ultrasound-guided biopsies are vital for confirming IBC and assessing nodal involvement.

In addition, contralateral axillary metastasis (CAM) is diagnosed in approximately 8.3% of IBC cases at presentation. CAM is best detected using ultrasound and PET^20^, with reported sensitivities and specificities as follows: mammography (18.2%/99.2%), ultrasound (92.3%/95.5%), PET (90.1%/99.1%), and MRI (76.0%/98.6%)^21^. Despite the importance of comprehensive nodal staging in IBC, studies have shown that only a minority of practitioners routinely obtain contralateral axillary imaging, and there is a lack of consensus regarding the surgical management of the axilla^22^.

Breast MRI can detect primary breast lesions in up to 100% of cases^19^, making it indispensable for assessing skin thickening, chest wall edema, and nodal involvement. MRI sensitivity increases by 35% with contrast^23^, and is considered the most sensitive tool for diagnosing multicentric disease^24^ and determining the extent of ipsilateral and contralateral involvement. Studies have also highlighted unique vascular patterns in IBC, such as increased angiogenesis, which may contribute to its aggressive behavior. Therefore, when you cannot accurately confirm the breast architectural changes by mammogram or breast US, we recommend ordering the Breast MRI mammogram.

Furthermore, pre-systemic therapy cross-sectional imaging (e.g. PET/CT, and CT scan of chest, abdomen, pelvis, bone scan) is essential for accurately mapping the anatomic distribution of regional nodal disease. This detailed anatomical information is critical for tailoring post-mastectomy radiation fields and adjusting radiation doses, ensuring a more precise and effective treatment strategy^25,26^. PET/CT remains a valuable modality for overall systemic staging due to its superior diagnostic performance compared to conventional imaging, particularly for detecting distant metastases^27^.

#### What is the role of diagnostic biopsy in identifying and managing IBC?

Diagnostic biopsy plays a crucial role in identifying IBC, particularly because traditional imaging often fails to reveal distinct masses or tumors, which are common in other forms of breast cancer. IBC typically presents with non-specific symptoms such as skin thickening, edema, or redness rather than a palpable mass. In these cases, core needle biopsies and skin punch biopsies are essential to obtain tissue samples from areas of abnormal skin (such as regions of erythema or peau d’orange) to confirm the presence of invasive carcinoma and rule out other causes of skin change^28^. An international expert panel on IBC, as outlined in a consensus statement for standardized diagnosis and treatment, strongly recommends core biopsy to confirm invasive IBC carcinoma. This procedure is also crucial for evaluating hormone receptor status (ER, PR) and HER2 expression, essential for guiding personalized treatment strategies^29^.

Biopsies are usually indicated when clinical suspicion for IBC is high, even without a detectable mass to assess the underlying pathology. The College of American Pathologists (CAP) Guidelines recommend biopsy procedures to ensure accurate reporting of the histologic grade, type, and lymphatic/vascular invasion, all of which are vital for guiding treatment strategies. The NCCN Guidelines also emphasize performing biopsies even when a mass is absent, ensuring accurate diagnosis and appropriate treatment planning. Adherence to these guidelines ensures that biomarkers such as ER, PR, and HER2 are properly assessed, allowing for precise treatment planning.

#### Is IBC genomically distinct from other types of breast cancer?

A comprehensive analysis of multiple studies has highlighted the complex genomic landscape and high mutational frequency characteristic of IBC. Mutational profiling has revealed that, when controlling for subtypes, mutations in pathways such as *NOTCH*, DNA repair, and *RAS/RAF* occur more frequently in IBC. Earlier studies identified frequent mutations in genes such as *TP53*, *PIK3CA*, and *ERBB2*, indicating their common occurrence in IBC, although these analyses did not always account for subtype-specific variations^30–32^. Since 2018, further research has emphasized the activation of *NOTCH*, DNA repair, and cell cycle pathways in IBC. For example, Liang et al. reported that 156 IBC cases exhibited higher mutation rates in these pathways than non-IBC, suggesting a distinctive genomic profile for IBC^33^. Similarly, Bertucci et al. analyzed 101 IBC cases. They found a median tumor mutational burden (TMB) of 6, compared to 2 in non-IBC, with significant differential expression of 96 genes, including those related to the *NOTCH* pathways and DNA repair mechanisms^34^.

Moreover, Richard et al. identified a high TMB and a significant rate of *AURKA* amplification (21%) in 34 IBC cases, alongside frequent alterations in *TP53* (65%) and *PIK3CA* (29%). However, these findings were insignificant in multivariable analyses, indicating the complexity of genomic influences on IBC^35^. In addition, Flávia L. C. Faldoni et al. described genomic alterations that contribute to the aggressiveness and metastatic potential of IBC, including *MDM4* gains and *CHL1* losses, along with high homologous recombination deficiency scores correlating with poorer OS and variants in homologous recombination and mismatch repair genes^36^.

Further studies by Li et al. and Priedigkeit et al., encompassing 20 and 140 cases, suggested that while IBC shares some genomic characteristics with non-IBC, such as comparable mutational burdens, IBC exhibits a higher incidence of *TP53* mutations. Importantly, these studies identified more frequent mutations in key pathways like *NOTCH*, DNA repair, and *RAS/RAF*, genomic differences between IBC and non-IBC continue to be influenced by receptor subtypes^37,38^. Additionally, the study identified significant differences in genomic alterations, gene expression, pathway enrichment, and immune cell levels between triple-negative IBC and triple-negative non-IBC. Specifically, triple-negative IBC exhibited a lower tumor mutation load and an association with immunosuppressive tumor-infiltrating immune components, which correlated with an unfavorable response to neoadjuvant chemotherapy^39^. Given IBC’s unique biological and clinical characteristics, routine DNA and RNA somatic sequencing can be justified in most, if not all, cases. IBC exhibits a distinct molecular profile compared to non-IBC breast cancers, characterized by higher frequencies of *TP53* mutations, alterations in the *PI3K/AKT* pathway, overexpression of *AXL* and *JAK/STAT* signaling, and an enrichment of epithelial-to-mesenchymal transition (EMT) and stem-like features. These molecular traits contribute to its aggressive behavior, early metastasis, and therapy resistance. Comprehensive genomic profiling provides critical insights into potential therapeutic targets, resistance mechanisms, and prognostic biomarkers. While targeted sequencing might suffice in other breast cancers, IBC’s heterogeneity and lack of standardized molecular subtyping call for a broader approach that includes RNA sequencing. RNA profiling is particularly valuable for capturing transcriptomic alterations, such as fusion genes immune signatures, and activating pro-metastatic pathways that may not be apparent from DNA sequencing alone. From a clinical trial perspective, integrating genomic profiling into routine practice can improve patient stratification, identifying those who might benefit from emerging targeted therapies, immunotherapy, or novel combination approaches. It also enables real-time adaptation of treatment strategies based on evolving tumor biology. Given the high recurrence rate and resistance to standard treatments in IBC, routine DNA/RNA somatic sequencing is strongly justified to guide treatment decisions at the first diagnosis, enhance clinical trial enrollment, and improve patient outcomes. Currently, no specific therapy is uniquely effective for IBC, underscoring the need for ongoing research to develop targeted treatments based on these genomic insights.

#### Why is a multidisciplinary approach crucial for accurate diagnosis of IBC?

A multidisciplinary approach is critical for improving overall survival (OS) for patients with IBC. Studies show that involving multiple specialties, such as oncology, surgery, pathology, and radiology, leads to better treatment coordination and significantly enhanced outcomes. For example, research from the Winship Cancer Institute revealed that metastatic IBC patients receiving comprehensive multidisciplinary care, including systemic therapy, surgery, and radiation therapy, had a median OS of 58 months compared to 19 months for those who did not receive surgical intervention^40,41^. Similarly, institutions like Johns Hopkins University, the Dana-Farber Cancer Institute, and The University of Texas MD Anderson Cancer Center emphasize that the complexity of IBC requires the combined expertise of oncologists, radiologists, and surgeons to create personalized treatment plans that improve outcomes.

Building on these findings, key stakeholders at The Ohio State University Stefanie Spielman Comprehensive Breast Center adopted a multidisciplinary approach using the Plan-Do-Study-Act model to develop an IBC program. Revising the call center decision tree reduced the median time to chemotherapy initiation to 10 days—a 43% decrease compared to previous years—and achieved 100% trimodality therapy among patients^42^.

Beyond accurate diagnosis, planning for post-treatment complications early in the process is crucial. While lymphedema^5^ is a well-known complication, many patients also experience severe cutaneous manifestations such as lymphangitic spread and Velpeau’s nodules—palpable, nodular skin lesions resulting from cancer cell dissemination through the dermal lymphatics. A rare but highly morbid complication of IBC is carcinoma en cuirasse, first described by Velpeau in 1883. Named for its resemblance to the steel breastplate of a cuirassier, carcinoma en cuirasse may present as an initial sign of primary disease. However, it more commonly occurs as a local relapse following mastectomy^43,44^. This sequela underscores the need for prompt, multidisciplinary input and ongoing treatment throughout the patient’s clinical course.

These complications can be as debilitating, or even more so, than lymphedema, warranting careful evaluation and management. Multidisciplinary teams must collaborate to manage these complex complications and ensure the best possible outcomes. Prospective assessment programs and pre-operative education also play a crucial role in patient care by allowing precise diagnoses and helping healthcare providers anticipate disease progression. Moreover, empowering patients with knowledge about their diagnosis and treatment options enables them to make informed decisions and actively participate in their care, enhancing satisfaction and improving long-term outcomes.

Collaboration among diverse professionals—including oncologists, surgeons, plastic surgeons, radiologists, pathologists, nurses, pharmacists, genetic counselors, nutritionists, and physical therapists—is essential for comprehensive care. Integrating multidisciplinary expertise streamlines the diagnostic and treatment process and lays a strong foundation for managing complex post-treatment challenges, ultimately improving patient outcomes in IBC.

#### What is the most effective form of trimodal therapy in treating IBC?

Advances in adjuvant therapy have significantly improved outcomes; however, these treatment options remain highly dependent on specific subtypes, mirroring approaches used in non-IBC. Given these nuances, personalized medicine is becoming essential for optimizing treatment in IBC^44–46^. Reflecting this shift, trimodal therapy, which combines systemic therapies such as chemotherapy, targeted therapy, and endocrine therapy (when appropriate) with local therapies like surgery and radiation, serves as a standard approach. This method is crucial for managing the disease’s local and metastatic stages^47^. A study involving 4374 patients who met the criteria for analysis revealed no significant association between neighborhood socioeconomic status and disease-specific mortality. However, notable disparities were observed with the triple-negative subtype (hazard ratio 2.66, 95% CI 2.21-3.20) and Black race (hazard ratio 1.41, 95% CI 1.16-1.72), both associated with higher disease-specific mortality compared to the luminal A subtype and White race, respectively. Notably, there was no difference in the receipt of trimodal treatment across different socioeconomic groups (P = 0.19)^48^.

Furthermore, a comprehensive review of over 10,000 IBC patients highlighted significant improvements in five-year and ten-year OS rates (55.4% and 37.3%, respectively) among those who received trimodal therapy. Despite these promising outcomes, trimodal therapy still needs to be utilized, with only about 40% of IBC patients completing the recommended treatment regimen as of 2014. Understanding and addressing the barriers to the full implementation of trimodal therapy could further improve survival outcomes for IBC patients^12^.

#### Does an anthracycline-sparing neoadjuvant regimen show promise in IBC?

Although anthracycline- and taxane-based chemotherapies are standards of care for IBC, concerns about anthracycline-related cardiotoxicity have prompted studies into anthracycline-sparing regimens^49^. However, the applicability of these results to the unique aggressiveness of IBC remains unclear, as these studies predominantly involved non-IBC patients. The BCIRG-006 trial^50^ showed comparable DFS and OS in early HER2+ breast cancer patients treated with both adjuvant anthracycline-containing and anthracycline-sparing regimens. Similarly, the TRYPHAENA trial showed equivalent pathological complete response(pCR) and DFS between these regimens when combined with dual HER2 blockade in a neoadjuvant setting^51^. These studies suggest that anthracycline-sparing regimens may benefit patients with HER2+ breast cancer by reducing the risks of cardiotoxicity and hematologic malignancies^52^.

Nevertheless, anthracycline- and taxane-based chemotherapy remains the standard recommendation for HER2 + IBC patients. In non-metastatic IBC, the typical approach is to administer anthracycline-based chemotherapy sequentially (to mitigate the cardiotoxicity associated with concurrent administration) followed by taxane-based chemotherapy combined with dual HER2 blockade (pertuzumab and trastuzumab) in the neoadjuvant setting. In contrast, for metastatic breast cancer, the standard first-line therapy is a taxane in combination with dual HER2 blockade rather than an anthracycline-containing regimen.

However, the treatment landscape is evolving in HER2-negative IBC cases. A joint analysis of the ABC trials by Blum et al., published in JCO 2017^53^, explored the efficacy of anthracycline-sparing regimens in HER2-negative patients. This study showed that non-anthracycline regimens, such as docetaxel plus cyclophosphamide, provided comparable efficacy to anthracycline-containing treatments in HER2-negative breast cancer. While this suggests potential for anthracycline-sparing regimens in HER2-negative IBC, further studies specific to IBC are needed to confirm their viability in this more aggressive subtype.

The use of non-anthracycline regimens is also gaining attention for triple-negative IBC. The Sharma et al. study, published in JAMA Oncology in 2024^54^, evaluated the combination of pembrolizumab with non-anthracycline chemotherapy in triple-negative breast cancer patients and demonstrated promising outcomes, including improved pCR rates. This suggests that anthracycline-sparing regimens, combined with immunotherapy, could offer a viable alternative for triple-negative IBC patients. Although this approach remains under investigation, it highlights the potential to reduce anthracycline-related toxicity without compromising outcomes.

Despite the global trend toward anthracycline-sparing regimens in breast cancer, we continue to recommend anthracycline- and taxane-based chemotherapy as the standard of care for IBC, particularly in the absence of robust, IBC-specific data supporting non-anthracycline alternatives. A pCR remains a crucial goal in IBC treatment, as it is strongly associated with improved long-term outcomes^55^. Therefore, until more IBC-specific data emerge, anthracycline-containing regimens should remain central to treatment strategies for this aggressive disease.

#### Can breast-conserving surgery or immediate reconstruction be performed instead of modified radical mastectomy in IBC?

With improvements in the pCR rates after NAC, the feasibility of breast-conserving surgery instead of modified radical mastectomy in IBC patients is being re-evaluated^56,57^. The ongoing ConSIBreC trial represents the first randomized clinical trial explicitly assessing the safety of breast-conserving surgery post-NAC in this patient population^58^. However, despite these developments, radical surgery remains the cornerstone of IBC treatment due to the lack of conclusive data supporting less invasive approaches. Current evidence does not recommend breast-conserving surgery in IBC due to concerns over local control and the aggressive nature of the disease.

Regarding reconstruction, primary breast reconstruction is not typically recommended for IBC patients. This is due to concerns that reconstruction may delay adjuvant therapies such as radiation and systemic treatments, which are critical for managing IBC. Instead, options like delayed reconstruction are often considered more appropriate, reflecting the radical nature of the required treatments and the need to prioritize timely adjuvant care. Long-term outcomes of delayed reconstruction appear similar, making it a more viable option in this aggressive disease context^59,60^.

#### What is the ideal radiation regimen in IBC?

For patients with IBC, postmastectomy radiation therapy to the chest wall and all regional lymph node basins is universally recommended^7^. Among the advanced strategies used, twice-daily radiation dose escalation^61^, and once-daily tissue bolus^62^ are notable. A retrospective study at MD Anderson Cancer Center showed that a dose escalation to 66 Gy twice daily significantly improved OS and locoregional control compared to 60 Gy in 115 non-metastatic IBC patients after modified radical mastectomy^61^. Similarly, a Mayo Clinic study reported 81% locoregional control at five years with once-daily bolus dosing of 60-66 Gy, with pCR showing a trend toward improved locoregional control^62^. These findings support the effectiveness of dose escalation and bolus dosing in achieving superior locoregional outcomes. However, treatments should be tailored to individual patient needs, especially for those who do not achieve a complete response to chemotherapy, as this appears to be associated with worse outcomes.

#### What is the ideal local therapy for de novo Stage IV IBC patients?

Treatment decisions for patients presenting with de novo Stage IV IBC should be highly individualized, taking into account factors such as tumor biology, disease extent, performance status, and patient preferences. The randomized clinical trial EA2108 demonstrated that early locoregional therapy for the primary site did not improve OS in patients with IBC and non-IBC metastatic breast cancer. However, it was associated with improved locoregional control^63^. For patients with IBC who have a life expectancy of greater than six months and can tolerate surgery and local radiation therapy, a multimodal approach—including systemic therapy followed by surgical resection and radiation—should be considered. Additionally, discussing Goal Concordant Care (GCC) with the patient, or if clinically indicated, with a patient representative, is critical in ensuring alignment with the patient’s preferences and overall treatment goals.

If a multidisciplinary evaluation concludes that the systemic therapy has elicited a good response and the patient is deemed operable, breast surgery and axillary lymph node dissection should be considered. In cases of extensive skin involvement, all grossly abnormal skin should be resected, and the patient will need plastic surgery assistance for chest wall closure or immediate lymphatic reconstruction.

In selected cases with limited metastatic spread and good performance status, aggressive local interventions, such as mastectomy (with or without axillary lymph node dissection) and radiation therapy, may be pursued with both palliative and potentially curative intent to control symptoms and improve survival^40,64^. Radiation therapy can also palliate painful or symptomatic metastases, such as those in bone or soft tissue, thereby improving local control and quality of life.

Clinical trials continue to explore the role of these therapies in managing metastatic IBC. Treatment planning should involve a multidisciplinary team—including medical oncologists, radiation oncologists, surgical oncologists, plastic surgeons, pharmacists, nurses, social workers, and other specialists—to ensure that each patient’s care is tailored to their specific clinical needs and goals.

#### What new therapeutic options are currently explored in ongoing clinical trials for IBC treatment?

Clinical trials play a crucial role in advancing the treatment of IBC, a notably aggressive form of the disease, with a dual focus on improving patient survival rates and preserving quality of life (QOL). A keyword search for “inflammatory breast cancer” on ClinicalTrials.gov^65^ returned 17 currently recruiting trials, of which only two are interventional drug studies specifically designed for IBC. In contrast, a broader review identified seven ongoing Phase II/III interventional trials that include patients with locally advanced or metastatic disease and do not exclude those with IBC (Table 2). While these trials are not exclusive to IBC, they may still offer potential therapeutic options. These trials explore novel drug treatments and combinations, reflecting a shift toward more personalized and effective therapies. This underscores the urgent need for innovative treatment approaches and highlights the challenges in developing effective therapies for this aggressive cancer.

**Table 2:** Ongoing Interventional Trials for Inflammatory Breast Cancer

Intervention	NCT#	Phase	Location	Type	Target
Trastuzumab deruxtecan and Durvalumab	05795101	II	US	Stage III, HER2 -, **IBC**	Tailoring neoadjuvant treatment
Trastuzumab Combined with Pyrotinib and Chemotherapy(docetaxel)	04481932	II	China	HER2 +, locally advanced	Tailoring neoadjuvant treatment
Carboplatin ± Tocilizumab	05846789	II	US	Triple Negative (TN) or ER low, Metastatic or locally recurrent	Treating metastasis
Pembrolizumab combined with Oral Metronomic Cyclophosphamide	03971045	II	Italy	locally recurrent, inoperable, and/or metastatic IBC involving the chest wall	Treating Advanced/ Metastasis
Seviteronel (administered with dexamethasone) in combination with chemotherapy (docetaxel)	04947189	I/II	Austraria	Androgen-receptor Positive, TN	Treating metastasis
Farletuzumab ecteribulin	04300556	I/II	Global (U.S. & Europe)	TN	Treating metastasis
Elacestrant + Everolimus	06382948	III	US	ER+/HER2-, ESR1mut, Progressing to ET and CDK4/6i	Treating Advanced/ Metastasis

Currently Recruiting Phase II and III Trials in Inflammatory Breast Cancer, Data retrieved from ClinicalTrials.gov on May 22, 2025.

Although IBC is often excluded from clinical trial eligibility, past trials such as BEVERLY-2, AZURE, and E2104 were designed in a way that could have allowed for the inclusion of IBC patients. For example, the BEVERLY-2 Trial, which aimed to evaluate the efficacy of adding bevacizumab, a VEGF inhibitor, to standard neoadjuvant chemotherapy in HER2-positive IBC patients, did not significantly improve pathological complete response rates or impact overall and event-free survival^66^. The AZURE Trial (BIG 01-04) assessed the addition of zoledronic acid to standard adjuvant therapy and found no significant improvement in disease-free survival (DFS) or OS, prompting a reassessment of bisphosphonates in adjuvant breast cancer treatment^67^. Lastly, the E2104 Trial tested the combination of thalidomide with chemotherapeutic agents in metastatic breast cancer, including IBC, but failed to achieve significant improvements in response rates or survival outcomes^68^. These past trials highlight the complexities of treating IBC and the difficulty in achieving significant outcomes across diverse patient populations.

Ongoing studies are now focusing on the efficacy of combining targeted therapies with standard chemotherapy to enhance treatment responses and reduce toxicity.

For example, ongoing studies are investigating the role of immunotherapy in combination with other modalities. The epidermal growth factor receptor (EGFR) expression in one-third of IBC cases correlates with poorer outcomes, prompting research into EGFR pathway blockade. Erlotinib, an EGFR tyrosine kinase inhibitor, has shown potential in suppressing cell proliferation in IBC cell lines, warranting further investigation^69^. Additionally, a Phase II study combining panitumumab, an anti-EGFR monoclonal antibody, with neoadjuvant chemotherapy reported a pathological complete response rate of 28% overall and 42% in the triple-negative subtype of IBC^70^. Despite these promising developments, panitumumab has not been established as a standard care due to insufficient data^71,72^.

Furthermore, immunotherapy shows significant promise in treatment IBC. Dual HER2-blockade, for example, has become the standard of care in neoadjuvant settings for HER2 + IBC, as affirmed by Neosphere^73^ and TRYPHAENA trials results^42^. The ongoing Phase II TRUDI trial explores the combination of Trastuzumab deruxtecan(T-DXd) with Durvalumab to enhance treatment efficacy in HER2 + IBC^74^ (Table 2). Several other trials are exploring anti-PD1/PDL1 immune checkpoint inhibitors, underscoring the potential of immunotherapies in IBC management^75^.

Understanding and addressing the barriers to fully implementing these promising new therapies could improve survival outcomes for IBC patients. Each ongoing trial aims to refine treatment strategies and ensure they are more widely applicable and effective across diverse patient populations.

#### How should we deal with lymphedema?

Breast cancer-related lymphedema (BCRL) is a debilitating complication that significantly impacts the QOL for breast cancer survivors, especially those undergoing treatment for IBC. Standard IBC treatment, which typically involves trimodal therapy—radical mastectomy, axillary lymph node dissection, and radiation therapy targeting the lymph nodes—dramatically increases the risk of BCRL. This condition can lead to lifelong impairment, serving as a persistent reminder of the cancer journey. Early intervention and management strategies are crucial for mitigating its long-term impact and improving patient outcomes^76^.

The risk of BCRL is significantly lower in patients who do not undergo radiation therapy, with approximately 1 in 5 breast cancer patients developing the condition^77^. However, in IBC patients receiving standard care, which includes aggressive treatments like radiation therapy, the risk rises dramatically, affecting around 50% of patients^5,78^. Contributing factors to BCRL include a body mass index (BMI) greater than 30 and non-white ethnicity, highlighting the importance of assessing these risks and implementing early lymphedema management strategies for at-risk populations^79,80^.

A proactive lymphedema screening program is essential, with adjustments to management as needed. This includes periodic replacement of compression sleeves every six months and referrals to Physical Therapy to improve range of motion. In some cases, referrals to Plastic Surgery should be made for procedures such as surgical lymphedema management.

Innovative surgical techniques that prevent or minimize lymphatic disruption, such as axillary reverse mapping (ARM) and lymphatic-venous anastomosis (LVA), show promise in reducing BCRL incidence. For patients unresponsive to conservative treatments like complete decongestive therapy (CDT), surgical interventions that aim to reduce limb volume or restore lymphatic flow may be necessary. A multidisciplinary approach to BCRL screening, treatment, and research is essential for providing comprehensive care for these patients.

The Lymphatic Microsurgical Preventive Healing Approach (LYMPHA), which bypasses transected lymphatics to alternate regions of outflow, has shown the potential to reduce lymphedema incidence in high-risk groups. Studies report a pooled cumulative incidence of 14.1% in patients undergoing axillary lymph node dissection (ALND), compared to 2.1% in those who underwent both ALND and LYMPHA (P = 0.029). In patients receiving ALND and regional lymph node radiation (RLNR), the incidence was 33.4%, which decreased to 10.3% when LYMPHA was added (P = 0.004). However, other studies suggest that LYMPHA may not significantly reduce lymphedema incidence in patients with obesity or those who have received radiation therapy, with no significant differences observed between LYMPHA and non-LYMPHA groups (P > 0.99)^77,81,82^.

While LYMPHA shows promise, more long-term studies are needed to fully understand its efficacy, particularly in patients undergoing ALND and radiation therapy. Alongside innovative approaches like ARM and LVA, future research will be crucial in determining whether LYMPHA can provide lasting lymphedema prevention, especially in high-risk populations receiving aggressive treatments.

#### What is the role of Goals of Care counseling in patient treatment plans?

Factors such as overall health, disease operability, life expectancy, and QOL shape treatment plans for patients with IBC. Goals of Care (GoC) counseling ensures treatment decisions align with the patient’s health status and personal preferences.

While a six-month prognosis often serves as a guideline for initiating hospice care in patients^83^, it is not a definitive timeline. Although hospice care is generally considered for those with a prognosis of six months or less—including IBC patients—individual responses to IBC can vary, and multiple factors may influence the duration of hospice eligibility.

For patients with a life expectancy of more than six months who can tolerate surgery, multimodal therapy—including surgical resection, local radiation, and systemic therapies—should be offered to extend life and maintain or improve QOL^84^. GoC discussions with the patient or caregiver help align these treatment plans with individual goals and expectations.

In contrast, for inoperable disease, systemic therapy, enrollment in clinical trials, and palliative radiation^85^ are advised to manage symptoms and preserve QOL. In these cases, GoC counseling ensures that treatment remains consistent with the patient’s preferences, focusing on comfort and QOL.

For patients with a life expectancy of less than six months or who cannot tolerate aggressive treatments, the focus should shift to systemic therapy, possible radiation, and symptom management. Regular GoC discussions allow for adapting care plans to the patient’s evolving needs, prioritizing QOL alongside clinical outcomes.

#### What are the health disparities present in patient care?

Health disparities present significant challenges in the treatment and outcomes of patients with IBC. There are several racial and socioeconomic disparities in the treatment and outcomes of patients with breast cancer. These disparities often stem from varied factors, including reduced access to specialized care, systemic mistrust, challenges in diagnosing conditions like erythema on darker skin, and biological differences across races/ethnicities^86^. For example, one study found that among IBC patients, a higher percentage of those with metastatic disease—compared to non-metastatic disease—were black, insured by Medicaid, and from areas of higher poverty and greater urban density^87^. In addition, black and Hispanic IBC patients experienced worse overall and breast cancer-specific survival compared to white patients, and those with Medicaid from urban areas or regions of higher poverty and lower education fared worse overall. Another study demonstrated that black women had poorer survival outcomes than non-Hispanic white women, regardless of inflammatory histology or hormone receptor status, while Asian/Pacific Islander women had better survival. These findings suggest that factors beyond histology and hormone receptor status likely contribute to racial/ethnic disparities in breast cancer survival^88^.

Moreover, the limited availability of recommended treatment protocols outside specialized high-volume cancer centers exacerbates these issues. There is a pressing need for enhanced education and awareness about IBC to combat these disparities, particularly in underserved communities. Consulting with an IBC specialist should be considered early when a diagnosis is suspected, and clinical trials should be made accessible to all patients to ensure equitable treatment opportunities. Increasing patient engagement and understanding through targeted educational initiatives can help bridge these gaps and improve overall outcomes in IBC care.

#### What surveillance should we provide post-treatment?

Comprehensive monitoring in IBC care goes beyond standard follow-up and involves an ongoing, multi-faceted approach that addresses physical and emotional aspects. This surveillance includes regular clinical evaluations to detect any signs of disease recurrence or progression and continuous management of treatment-related side effects^89^. Interprofessional care is essential, with targeted education programs, prospective lymphedema screening, and pre-operative education being key components for effective management. Regular assessments of lifestyle risk factors, body image concerns, and psychosocial support also form part of this comprehensive approach, given the significant impact of IBC treatments on patients’ quality of life and mental well-being.

Lifestyle interventions are vital for improving outcomes. Research suggests that maintaining an active lifestyle, following a healthy diet, limiting alcohol intake, and achieving an ideal body weight (BMI between 20 and 25) can lead to better breast cancer outcomes. These practices are key to enhancing survival and quality of life after treatment.

Effective communication is critical in coordinating care between primary care providers and specialists. Offering patients a personalized survivorship plan, which includes a detailed treatment summary, information on potential long-term toxicities, and follow-up recommendations, ensures comprehensive and consistent care.

Engaging patients is equally important, as they often need encouragement to adhere to follow-up screenings and medication regimens. Continued support from healthcare providers improves adherence and improves long-term health outcomes. Additionally, comprehensive monitoring incorporates genetic counseling, fertility preservation, and family planning discussions, ensuring that the long-term effects of treatment on reproductive health are addressed. This holistic care framework is essential for patients whose treatment may impact future family planning decisions. By integrating physical, emotional, and genetic monitoring elements alongside lifestyle management, effective communication, and patient engagement, comprehensive care enhances IBC survival and optimizes patients’ quality of life after treatment.

#### What are the future and potential innovations in the treatment of IBC?

Recent advancements in diagnostic methodologies, including developing a new scoring system, have begun to provide more precise definitions, thus aiding in the more accurate identification of IBC. However, robust data to support the de-escalation of surgical therapy for IBC remains lacking^90^. Trimodality therapy- which consists of neoadjuvant systemic therapy, modified radical mastectomy, and post-mastectomy radiation- continues as the standard treatment approach, having significantly improved survival rates over the past four decades. In addition, HER2-targeted therapies - such as dual blockade with pertuzumab and trastuzumab and, in appropriate settings, adjuvant T-DM1- have become a cornerstone of treatment, resulting in improved pathologic complete response rates and better overall outcomes.

Immunotherapy has emerged as a game-changer, particularly in triple-negative breast cancer, and its benefits extend to IBC patients. The KEYNOTE-522 trial, which included a subgroup of patients with T3/T4 tumors—demonstrated significant improvements in pCR and survival outcomes by adding pembrolizumab to neoadjuvant chemotherapy. Similarly, the KATHERINE trial showed that adjuvant T-DM1 provided benefits in patients with residual disease, including those with T4d tumors. Additionally, the OlympiA trial and the Create-X study further support the value of integrating novel systemic therapies into IBC treatment. Moreover, pivotal trials such as NOHA and TRYPHAENA have provided valuable insights into dual HER2 blockade in IBC, underscoring those treatments like pembrolizumab and the combination of trastuzumab/pertuzumab are established treatments.

Looking ahead, novel antibody-drug conjugates (ADCs) such as T-DXd and sacituzumab govitecan are emerging as powerful agents that may further revolutionize IBC management. These drugs have demonstrated impressive efficacy in advanced breast cancer and are expected to have a significant impact on treatment paradigms in IBC

Furthermore, integrating immunotherapies and targeted therapies, particularly those targeting EGFR, VEGF, and other key pathways—is a present reality with substantial patient benefits. Despite the promising potential of these therapies, the rarity of IBC often leads to underrepresentation in more extensive breast cancer clinical trials, complicating the analysis of treatment efficacy specifically for IBC^4^. This highlights the urgent need for clinical trials focused exclusively on IBC to overcome misclassification and underpowered studies that make evaluating therapeutic efficacy challenging. A key consideration is whether IBC is genomically distinct from other breast cancer subtypes. Some studies have reported more frequent mutations in pathways such as NOTCH, DNA repair^34^, and RAS/RAF^33^, along with unique interactions within the tumor microenvironment, including the involvement of inflammatory pathways identified through gene expression signatures^91^. However, no single gene expression signature consistently identifies IBC cases, and there is little overlap between the various signatures that have been identified.

Emerging research on circulating tumor DNA (ctDNA) has shown promise, with studies indicating its potential to identify actionable genomic abnormalities and monitor treatment efficacy in real time^92,93^. An observational analysis of 35 IBC patients demonstrated the utility of ctDNA in guiding IBC management. In comparison, a retrospective review of 18 patients revealed that the presence of ctDNA after surgery was associated with poorer survival outcomes, underscoring its potential as a critical biomarker in IBC treatment^94^. Given these findings, ctDNA could become a valuable tool for monitoring IBC patients, allowing for dynamic adjustments in therapy based on molecular insights. These innovations are poised to significantly influence future treatment paradigms, emphasizing the need for ongoing research and the development of tailored therapeutic strategies specific to IBC.

#### Should patients need to see a highly specialized IBC clinic if diagnosed with IBC?

Given the aggressive nature of IBC and the necessity for a multidisciplinary approach, patients diagnosed with IBC should ideally be referred to a specialized IBC clinic. These clinics provide “one-stop” patient visits, advanced expertise in diagnosing and treating IBC, access to clinical trials, and the latest treatment protocols, all of which can significantly enhance patient outcomes. However, barriers such as insurance limitations, financial constraints, or geographic distance may prevent many patients from accessing these specialized centers. In such cases, alternative strategies should be considered.

When direct access to an IBC clinic is not feasible, community healthcare providers can consult with IBC specialists through telemedicine or other forms of remote consultations. Another role of IBC clinics includes providing virtual visits for rural or geographically isolated patients, as well as outreach and training for medical staff. Following national guidelines, such as those from the NCCN, and reviewing specific institutional protocols, like the MD Anderson Cancer Center’s IBC treatment guidelines available online, can help maintain best practices. However, implementing MD Anderson’s protocols in community settings may pose challenges due to limited resources and a lack of specialized care physicians.

In these situations, providers should consider utilizing local multidisciplinary teams experienced in advanced breast cancer care and exploring available clinical trial options for the patient. Staying informed about the latest treatment recommendations, taking advantage of patient assistance programs, and collaborating with cancer care navigators can further help reduce barriers to ensure patients receive timely, evidence-based care, even in resource-limited settings.

## Conclusions

Inflammatory breast cancer remains an aggressive disease that presents substantial challenges in treatment. This manuscript provided foundational knowledge to identify critical areas requiring attention, enabling best practices in real-world settings, especially where IBC specialists may not always be available. As advancements continue in breast cancer treatment, it is essential to assess their relevance and applicability to IBC to ensure patients benefit from the latest therapeutic innovations. Expanding our understanding of the unique biological mechanisms underlying IBC holds promise for significantly improving survival rates by incorporating targeted therapies into treatment regimens. The IBC diagnostic scoring system, recently validated using a large multi-institutional clinical database, is a groundbreaking tool that improves diagnostic accuracy and supports treatment planning, and may reduce delays in care-particularly in settings where specialist input is limited.

Participation in clinical trials is essential, but such opportunities are often unavailable or infeasible. Guidelines are crucial because they reflect recent therapeutic advancements and address the complexities involved in surgery, radiation, and chemotherapy. We should prioritize strategies that enhance patients’ quality of life. A comprehensive, multidisciplinary approach is vital for achieving better outcomes in this challenging area of oncology. That said, pragmatic approaches are needed to recommend diagnostic tools or treatments that are not feasible due to resource limitations.
